# Noncommunicable diseases risk factors in Bhutan: A secondary analysis of data from Bhutan’s nationwide STEPS survey 2014

**DOI:** 10.1371/journal.pone.0257385

**Published:** 2021-09-23

**Authors:** Gyambo Sithey, Li Ming Wen, Laigden Dzed, Mu Li

**Affiliations:** 1 Centre for Research Initiatives, Changangkha, Thimphu, Bhutan; 2 Sydney School of Public Health, The University of Sydney, Sydney, NSW, Australia; 3 Population Health Research and Evaluation Hub, Sydney Local Health District, Sydney, NSW, Australia; 4 Nutrition Program, Department of Public Health, Ministry of Health, Thimphu, Bhutan; University of Mississippi Medical Center, UNITED STATES

## Abstract

**Background:**

Bhutan is facing an epidemic of noncommunicable diseases; they are responsible for 53% of all deaths. Four main modifiable risk factors, including tobacco use, harmful use of alcohol, physical inactivity, and unhealthy diet, are the causes of most noncommunicable diseases (NCDs). This study aimed to assess 1) the prevalence of NCDs modifiable risk factors in Bhutan’s adult population and 2) associations between the sociodemographic factors and the NCDs modifiable risk factors with overweight or obesity, hypertension, and diabetes.

**Methods:**

We used the 2014 Bhutan WHO Stepwise Approach to NCD Risk Factor Surveillance (STEPS) Survey dataset in this study. Data were analyzed using multiple logistic regressions, constructed with overweight or obesity, hypertension, and diabetes as outcome variables and modifiable risk factors as independent variables.

**Results:**

The prevalence of tobacco use, harmful use of alcohol, unhealthy diet (low fruits and vegetables intake) and physically inactive was 24.8% (95% CI: 21.5, 28.5), 42.4% (95% CI: 39.4, 45.5), 66.9% (95% CI: 61.5, 71.8), and 6.2% (95% CI: 4.9, 7.8), respectively. The prevalence of overweight or obesity, hypertension and diabetes was 32.9% (95%CI: 30.0, 36.0), 35.7% (95% CI: 32.8, 38.7) and 6.4% (95% CI: 5.1, 7.9), respectively. Multiple logistic regression showed that older age groups were more likely to be overweight or obese, hypertensive, and diabetic. Our analysis also found that tobacco users were less likely to be overweight or obese (aOR 0.71, 95% CI 0.52, 0.96), and to be hypertensive (aOR 0.74, 95% CI 0.56, 0.97); but they were more likely to be diabetic (aOR 1.64, 95% CI 1.05, 2.56). Alcohol users were more likely to be hypertensive aOR 1.41 (95% CI 1.15, 1.74). Furthermore, vigorous physical activity could protect people from being overweight or obese, aOR 0.47 (95% CI 0.31, 0.70), and those consuming more than five serves of fruits and vegetables per day were more likely to be overweight or obese, aOR 1.46 (95% CI 1.17, 1.82).

**Conclusion:**

The prevalence of NCDs modifiable risk factors and overweight or obesity and hypertension was high in Bhutan. We found strong associations between tobacco use and diabetes, alcohol use, hypertension, physically inactive, and overweight or obesity. The results suggest that the government should prioritize NCDs prevention and control programs, focusing on reducing modifiable risk factors. The health sector alone cannot address the NCDs epidemic in Bhutan, and we recommend the whole of government approach to tackle NCDs through the Bhutan Gross National Happiness framework.

## Introduction

Noncommunicable diseases are the leading cause of death worldwide. Each year about 41 million lives are lost to NCDs or 70% of all deaths globally [1]. Almost 80% of all NCDs deaths occur in low- and middle-income countries [1]. The World Economic Forum estimated that the cost of under-investment in the fight against NCDs would be in the order of US$ 47 trillion in lost gross domestic product globally from 2011 to 2025, forcing millions of people to continue living below the poverty line [2]. The four most common modifiable behavioral risk factors for NCDs are tobacco use, harmful alcohol use, physical inactivity, and unhealthy diet [3]. It is estimated that more than half of the NCDs burden could be avoided by preventing these four modifiable behavioral risk factors [4].

Bhutan is a small landlocked Himalayan country and one of the least developed countries based on the United Nations’ the least developed countries list (as of February 2021) [5]. Seventy percent of the Bhutanese population resides in rural areas, and 62.2% of the population’s livelihoods are involved in agriculture [6]. While Bhutan has adopted a cautious economic development, it faces an epidemiological transition and the NCDs epidemic. Sixty-nine percent of deaths and 23% of projected premature mortality were from NCDs in 2016 [1]. The growing epidemic of NCDs threatens to undermine the achievement of Bhutan’s Gross National Happiness [7]. Gross National Happiness is a developmental philosophy adopted by Bhutan, which aims to achieve a holistic, sustainable, and balanced form of development by considering a range of domains, each of which makes a vital contribution to happiness. Health is one of the domains. Therefore, investigating the magnitude of NCD risk factors and their adverse health outcomes will help Bhutan develop prevention strategies and interventions to reduce morbidity and mortality from NCDs and promote population well-being.

The paper aimed to estimate the prevalence of modifiable risk factors for NCDs in Bhutan and investigate the associations of sociodemographic factors and modifiable risk factors with overweight or obesity, hypertension, and diabetes, respectively. The findings from this study could be helpful to formulate NCDs prevention and control strategies in Bhutan.

## Materials and methods

### Study design

This study was a secondary analysis of data extracted from the first nationally representative cross-sectional STEPS survey for NCDs risk factors conducted by the Ministry of Health, Royal Government of Bhutan in 2014 using the WHO STEPS protocol [8].

### Sampling

In this national survey, a multi-stage sampling methodology was used to select enumeration areas, households, and respondents. The sample size was calculated based on the prevalence of overweight or obesity (53%) from the 2007 STEPS survey conducted in Thimphu, the capital city. A total of 182 secondary sampling units were selected initially through the probability proportionate to size method. From each secondary sampling unit, 16 households were chosen using systematic random sampling. Finally, using the Kish sampling method to select one eligible household member at the household level randomly. In total, 2912 respondents aged 18–69 years were sampled, assuming an 80% response rate. Further details of the data collection and management procedures are described in the National survey for noncommunicable diseases risk factors and mental health using the WHO STEPS approach in Bhutan-2014 [9].

### Data collection

Data collection followed the core STEPS protocol: STEP I included information on socio-demographics (i.e., age, sex, education, marital status, area and occupation) and modifiable risk factors namely, tobacco use, harmful consumption of alcohol, fruits and vegetables consumption, and level of physical activity. STEP II covered physical measurements including height, weight, waist circumference and blood pressure; STEP III biological measurements including fasting blood glucose and total cholesterol level [8]. Blood total cholesterol level was not measured in this survey due to technical constraints.

### Measures used in this study

#### Modifiable behavioral risk factors

Tobacco use was defined as the current use of smoked and/or smokeless (chewing) forms of tobacco. Alcohol use was defined as individuals who consumed alcohol within the past 30 days [9]. Physical activity was classified into three groups. The high and moderate physical activity were defined as seven or more days of any combination of walking, moderate or vigorous-intensity activities accumulating at least 3000 MET-minutes/week, and five or more days of any combination of walking, moderate-intensity or vigorous intensity activities achieving a minimum 600 MET-minutes/week, respectively. Low physical activity was defined as MET-minutes/week <600MET in a form of work situation, travel from place to place, or recreational activities. [10, 11]. Unhealthy diet, i.e. low fruits and vegetables intake was defined as < 5 servings per day [10].

#### Study outcome measures overweight or obesity, hypertension, and diabetes

Overweight or obesity was defined as BMI ≥ 25.0 kg/m^2^. Hypertension was defined as systolic blood pressure ≥140 mm Hg and/or diastolic blood pressure ≥90 mm Hg during the survey or on medication for raised blood pressure. Diabetes was defined as individuals with a fasting blood sugar of ≥ 126 mg/dL [8] or on medication for diabetes.

### Data analysis

We extracted data from the STEPS survey dataset and imported it to Stata version 13, which was used for all analyses (Stata Statistical Software: Release 13 College Station, TX: StataCorp LP). Separate weights were calculated for the three different steps because the sample size variation in the three steps due to a different response rate in each step. For example, the response rate was 96.9% in STEP I, 96.7% in STEP II, and the response rate was 93.5% in STEP III for blood measurements and 89.9% for urine collection.

All estimates were weighted and presented with percentages and odds ratios, including 95% confidence intervals (CI).

The potential risk factors for NCDs were sociodemographic factors (including age, sex, education, marital status, area of living, and occupation) and the four common modifiable behavioral risk factors. The study outcome factors included overweight or obesity, hypertension, and diabetes.

In the first step, we examined the characteristics of the respondents by sex using the Chi-squared test. In the second step, bivariate associations between the outcome variables (i.e., overweight or obesity, hypertension, and diabetes) and categorical risk factors were also examined by the Chi-squared test. Finally, multiple logistic regression models were constructed in the third step with overweight or obesity, hypertension, and diabetes as outcome variables [8]. The outcome variables were modeled individually with sociodemographic and modifiable behavioral risk factors as the potential independent variables. All variables associated with outcomes at p ≤0.25 in the univariate analyses were included in multiple logistic regression models to allow for maximum potential confounders included in the model [12]. We then employed backward elimination stepwise processes and eliminated non-significant variables (p >0.05) from the model, assuming a lack of confounding if the parameter estimate of all remaining variables did not change by more than 10% after addition or removal of the potential confounder. The odds ratio was calculated using the model reflects the likelihood of having one of the study outcomes (i.e., overweight or obesity, hypertension, and diabetes). Adjusted Odds Ratio (aOR) and 95% CI were used to interpret the adjusted risk, and p < 0.05 was considered statistically significant. Collinearity between variables was checked using variance inflation factors and found to be < 2 [13]. All analyses, univariate and multivariate logistic regression, were adjusted for cluster and sample weights.

### Ethics statement

For this study we used the 2014 Bhutan STEPS survey dataset with all information unidentifiable; ethics approval was not required. We obtained permission for using the dataset to conduct the secondary data analysis from the Secretariat, Ministry of Health, Royal Government of Bhutan.

## Results

### Characteristics of survey participants

The sociodemographic characteristics of the survey population are presented in Table 1. In total, 2822 adults (18–69 years) participated in the survey. Of these, 1074 (38.1%) were men and 1748 (61.9%) were women, 68.6% were from rural areas, 56.1% had no formal education (not attended monastic institution or modern school), 82.7% were married, 53.1% were self-employed mainly farmers, 22.9% were government employees and 14.1% were homemakers. Overweight or obesity, hypertension and diabetes were measured in 2748 (97.3%), 2814 (99.7%), and 2743 (97.2%) respondents, respectively, of the total 2822 respondents of the survey questionnaire.

**Table 1:** Characteristics of the survey respondents and the prevalence of risk factors by sex.

Risk factors		Total N (%)	Men n (%)	Women n (%)
		2822	1074 (38.1)	1748 (61.9)
Age				
	18–24 years	281 (13.0)	78(10.7)	203 (15.9)
	25–34 years	761 (34.1)	264(35.4)	498 (32.5)
	35–44 years	751 (26.1)	284(26.7)	467 (25.3)
	45–54 years	572 (14.4)	239(14.4)	333 (14.5)
	55–69 years	456 (12.4)	209(12.8)	247 (11.8)
Education^1^				
	No formal education	1766 (56.1)	574(48.1)	1192 (66.3)
	Formal Education	1054 (43.9)	499(51.9)	555 (33.7)
Marital Status				
	Married	2278 (82.7)	907(84.5)	1371 (80.5)
	Never married	225 (10.5)	104(12.0)	121 (8.6)
	Single/divorce/widow	317 (6.8)	62 (3.5)	255 (10.9)
Area of living				
	Rural	1952 (68.6)	775 (70.3)	1177 (66.6)
	Urban	870 (31.4)	299 (29.7)	571 (33.4)
Occupation				
	Self-employed	1558 (53.1)	606(53.6)	952 (52.6)
	Homemakers	559 (14.1)	26 (1.5)	533 (30.2)
	Government	480 (22.9)	328(33.5)	152 (9.4)
	Others	223 (9.8)	113(11.4)	110 (7.9)
Tobacco use^2^				
	Non users *	2255 (75.2)	733 (66.4)	1522 (86.4)
	Users	565 (24.8)	340 (33.6)	225 (13.6)
Alcohol use^3^				
	Non users *	1685 (57.6)	527 (50.0)	1158 (67.2)
	Users	1164 (42.4)	574 (50.0)	590 (32.8)
Physical activity^4^				
	Inactive*	204 (6.2)	53 (3.8)	151 (9.2)
	Moderate	408 (12.8)	126 (10.3)	282 (15.9)
	Vigorous	2196 (81.0)	889 (85.9)	1307 (74.8)
Dietary habits^5^				
	< 5serving/day**	1901 (66.9)	702 (64.8)	1199 (69.6)
	≥ 5 servings/day	916 (33.1)	34.5 (35.2)	547 (30.4)

*p≤0.05

** p>0.05.

^1^Formal education referring to ever enrolled in either a monastic institution or a school.

^2^Users including smoked and smokeless.

^3^Users including any alcohol consumption within the past 30 days.

^4^Inactive (<600 MET minutes per week), Moderate (600–2999 MET minutes per week), Vigorous (≥ 3000 MET minutes per week).

^5^Servings of fruits and vegetables on average per day.

### Prevalence of modifiable risk factors

The overall prevalence of current tobacco use (smoked or smokeless form) was 24.8% (95% CI: 21.5, 28.5), 7.4% were smoking, and 19.7% were using smokeless (chewing) tobacco, with some using both forms. Tobacco use was significantly higher in men 33.6% (95% CI: 28.9, 38.7) than women 13.6% (95% CI: 11.1, 16.6) (p <0.05). The overall prevalence of current alcohol use (in the last 30 days) was 42.4% (95% CI: 39.4, 45.5). More men [50% (95%CI: 45.5, 54.4)] were consuming alcohol than women [32.8% (95% CI: 29.6, 36.1) (p <0.05)]. The overall prevalence of low physical activity was 6.2% (95% CI: 4.9, 7.8). The prevalence was significantly higher in women [9.2% (95% CI: 7.0, 12.1)] than men [3.8% (95% CI: 2.7, 5.1) (p<0.05)]. The prevalence of low physical activity was significantly higher (p <0.05) in urban areas [12.9% (95% CI: 9.2, 17.7)] than the rural areas [3.1% (95% CI: 2.3, 4.2)]. Overall, 66.9% (95%CI: 61.5, 71.8) of the population had low fruits and vegetables intake, which was not significantly different by sex (Table 1). There was no significant difference in tobacco use, current alcohol use, and low fruits and vegetables intake by the living area.

### Prevalence of overweight or obesity, hypertension, diabetes

The overall prevalence of overweight or obesity (BMI ≥ 25) was 32.9% (95% CI: 30.0, 36.0). There was a significant difference in the prevalence of overweight or obesity by sex, age, marital status, area of living, occupation and by modifiable risk factors like tobacco use and levels of physical activity (p <0.05) (Table 2). The overall prevalence of hypertension was 35.7% (95% CI: 32.8, 38.7). There was a significant difference in the prevalence of high blood pressure by age, education, marital status, occupation, or modifiable behavioral risk factors, including tobacco use and alcohol use (p<0.05). However, there was no significant difference in the prevalence of high blood pressure by sex, area of living, levels of physical activity and dietary habits (Table 2). The overall prevalence of diabetes was 6.4% (95% CI: 5.1, 7.9). There was a significant difference in the prevalence of diabetes by sex, area of living, and age (Table 2).

**Table 2:** Bivariate analysis of factors associated with overweight or obesity, hypertension, diabetes.

Risk factors	Total N = 2822	Overweight/ Obesity^6^ n (%)	Hypertension^7^ n (%)	Diabetes^8^ n (%)
Sex				
	Men	321 (27.4)*	423 (35.5)**	73(6.5)*
	Women	730 (40.3)	678 (35.9)	110 (6.3)
Age				
	18–24 years	45 (14.1)*	34(13.0)*	8(3.9)*
	25–34 years	289 (33.3)	191(25.2)	27(2.8)
	35–44 years	309 (36.1)	319 (40.4)	50(9.4)
	45–54 years	240 (39.3)	287 (51.5)	52(10.0)
	55–69 years	168 (36.8)	270 (60.2)	46(8.4)
Education^1^				
	No formal education	660 (32.9)**	783(40.6)*	125(6.9)**
	Formal Education	391 (33.0)	318 (29.5)	58(5.8)
Marital Status				
	Married	898 (35.0)*	906 (36.9)*	149(6.6)**
	Never married	33 (12.9)	42 (19.6)	10 (4.1)
	Single/divorce/widow	120 (38.5)	153 (46.1)	24 (7.2)
Area of living				
	Rural	630 (27.5)*	778 (35.9)**	112 (5.4)*
	Urban	421 (45.0)	323 (35.4)	71 (8.6)
Occupation				
	Self-employed	555 (28.4)*	661(39.0)*	94 (5.1)**
	Homemakers	271 (50.4)	184 (31.5)	37 (6.8)
	Government Employees	187 (40.3)	177 (33.6)	39 (8.5)
	Others	38 (16.1)	79 (28.7)	13 (8.0)
Tobacco use^2^				
	Non users	890 (35.8)*	907 (37.5)*	141 (5.8)**
	Users	161 (24.4)	194 (30.4)	42 (8.2)
Alcohol use^3^				
	Non users	608 (31.8)**	576 (31.9)*	99 (6.2)**
	Users	443 (34.5)	525 (40.9)	84 (6.8)
Physical activity^4^				
	Inactive	110 (54.8)*	84 (39.4)**	12 (5.5)**
	Moderate	179 (45.0)	161 (39.1)	33 (7.3)
	Vigorous	756 (29.3)	853 (35.0)	137 (6.3)
Dietary habits^5^				
	< 5serving/day	665 (30.7)**	711 (34.3)**	125 (6.6)**
	≥ 5 servings/day	386 (37.4)	389 (38.6)	58 (6.1)

*p≤0.05

** p>0.05.

^1^Formal education referring to ever enrolled in either a monastic institution or a school.

^2^Users including smoked and smokeless.

^3^Users including any alcohol consumption within the past 30 days.

^4^Inactive (<600 MET minutes per week), Moderate (600–2999 MET minutes per week), Vigorous (≥ 3000 MET minutes per week).

^5^Servings of fruits and vegetables on average per day.

^6^Overweight/obesity = BMI ≥25 kg/m^2^ and non pregnant.

^7^Hypertension = SBP > 140 mm Hg or DBP > 90 mm Hg or on medication for hypertension.

^8^Diabetes = Fasting blood glucose ≥ 126 mg/dL and/or on medication for diabetes.

### Independent risk factors for overweight or obesity, hypertension, diabetes

#### Overweight or obesity

Results of the multiple logistic regression analysis (Table 3) showed associations between overweight or obesity with age, sex, marital status, area of living, occupation, tobacco use, level of physical activity, and dietary habits after adjusting for education and alcohol use (p <0.05). Compared to the 18–24-year-olds group, older age groups were more likely to be overweight or obese. Further, being female, single/divorced/widow, homemakers or government employees, living in urban areas, and consuming ≥ five servings of fruits and vegetables per day were more likely to be overweight or obese. Tobacco use and being moderately active and vigorously active physically were less likely to be overweight or obese.

**Table 3:** Independent risk factors for overweight or obesity, hypertension or diabetes using multiple logistic regression models.

		Overweight or obesity^6^		Hypertension^7^		Diabetes^8^	
		aOR^9^ (95% CI)	P value	aOR (95% CI)	P value	aOR (95% CI)	P value
*Sociodemographic characteristics*							
Age			<0.001		<0.001		<0.001
	18–24 years	Referent		Referent		Referent	
	25–34 years	2.66 (1.69, 4.18)		2.01 (1.21, 3.35)		0.82 (0.31, 2.18)	
	35–44 years	3.41 (2.28, 5.10)		4.08 (2.46, 6.76)		3.24 (1.34, 7.84)	
	45–54 years	4.09(2.60, 6.finan43)		6.17 (3.66, 1.04)		4.02 (1.62, 9.99)	
	55–69 years	4.11(2.66, 6.33)		9.23 (5.61, 15.19)		3.44 (1.43, 8.32)	
Sex			<0.001				
	Men	Referent		Referent		Referent	
	Women	1.70 (1.27, 2.28)		a^10^		a	
Education^1^					0.996		
	No formal education	Referent		Referent		Referent	
	Formal education	a		1.0 (0.76, 1.33)		a	
Marital Status			0.039		0.834		
	Married	Referent		Referent		Referent	
	Never married	0.54 (0.34, 0.87)		0.96 (0.58, 1.61)		a	
	Single/divorce/widow	0.99 (0.74, 1.33)		1.10 (0.80, 1.42)		a	
Area of living			<0.001				0.021
	Rural	Referent		Referent		Referent	
	Urban	1.79 (1.38, 2.32)		a		1.74 (1.09, 2.79)	
Occupation			<0.001		0.680		0.242
	Self-employed	Referent		Referent		Referent	
	Homemaker	1.81 (1.29, 2.56)		0.87 (0.65, 1.17)		1.46 (0.78, 2.75)	
	Government employee	1.63 (1.18, 2.26)		1.05 (0.70, 1.58)		1.63 (0.98, 2.73)	
	Others	0.76 (0.39, 1.51)		0.95 (0.61, 1.50)		1.95 (0.65, 5.80)	
*Modifiable risk factors*							
Tobacco use^2^			0.025		0.030		0.030
	Non users	Referent		Referent		Referent	
	Users	0.71 (0.52, 0.96)		0.74 (0.56, 0.97)		1.64 (1.05, 2.56)	
Alcohol use^3^			0.084		0.001		
	Non users	Referent		Referent		Referent	
	Users	1.21 (0.97, 1.50)		1.41 (1.15, 1.74)		a	
Physical activity^4^			<0.001				
	Inactive	Referent		Referent		Referent	
	Moderate activity	0.74 (0.48, 1.16)		a		a	
	Vigorous activity	0.47 (0.31, 0.70)		a		a	
Dietary habits^5^			0.001		0.197		
	< 5 servings fruits and vegetables per day	Referent		Referent		Referent	
	≥5 servings of fruits and vegetables per day	1.46 (1.17, 1.82)		1.21 (0.90, 1.62)		a	

^1^Formal education referring to ever enrolled in either a monastic institution or a school.

^2^Users including smoked and smokeless.

^3^Users including any alcohol consumption within the past 30 days.

^4^Inactive (<600 MET minutes per week), Moderate (600–2999 MET minutes per week), Vigorous (≥ 3000 MET minutes per week).

^5^Servings of fruits and vegetables on average per day.

^6^Overweight/obesity = BMI ≥ 25 kg/m^2^.

^7^Hypertension = SBP > 140 mm Hg or DBP > 90 mm Hg or on medication for hypertension.

^8^Diabetes = Fasting blood glucose ≥ 126 mg/dl and or on medication for diabetes.

^9^aOR: adjusted for other variables in the table.

^10^ Not applicable.

#### Hypertension

Although hypertension was significantly associated with age, tobacco use, and alcohol use (p <0.05), it was not significantly associated with sex, education, marital status, occupation, physical activity levels, and dietary intake of fruits and vegetables. When using 18–24 years as the reference category, there was a significant increase in the odds of reported hypertension with age. Among the modifiable behavioral risk factors, tobacco use was less likely to be associated with hypertension, while current alcohol use was more likely to be associated with hypertension (Table 3).

#### Diabetes

Diabetes was significantly associated with age, area of living, and tobacco use. Compared to those aged 18–24 years, people in older age groups were more likely to be diabetic. Similarly, urban residents and tobacco users were also more likely to be diabetic. Sociodemographic factors like sex, marital status, education, occupation, and modifiable behavioral risk factors such as alcohol, low physical activity, and dietary habits were not significantly associated with diabetes (Table 3).

## Discussion

This study showed that overweight or obesity and hypertension were significant public health problems in Bhutan, with about one-third of the adult population being overweight or obese (32.9%) and hypertensive (35.7%). There was a wide variation in the prevalence of NCDs modifiable risk factors by age, sex, and living area. Hence, routine and periodic national screening and surveillance for NCDs should be integrated into primary health care for early detection and management.

Although the cultivation, manufacture, sale, and distribution of tobacco products have been banned in Bhutan since June 2010 [14], the prevalence of tobacco use was high (24.8%), and the majority of users consumed smokeless tobacco predominantly (19.7%). Gurung et al. [15] attributed the higher proportion of smokeless tobacco use to its affordability. The national ban on the import and sale of tobacco has pushed the illegal sale of tobacco products to an exorbitant price. As a result, a much higher prevalence of smokeless tobacco consumption than smoking has been reported in Bhutan [16]. Further, the smoking ban in public places and random inspections by authorities might have deterred people from smoking.

The annual per capita adult alcohol consumption (8.5 liters) in Bhutan is substantially higher than the average global consumption (6.2 liters) [17]. The government’s decision to relax restrictions on the production and distribution of alcoholic beverages, including bar licenses and low cost of alcohol, coupled with cultural norms of alcohol consumption, are part of the explanations of the high prevalence of alcohol use [17]. Culturally, alcohol is a vital part of religious ceremonies and social functions. Therefore, it is common for Bhutanese to drink; some started at a very young age [17, 18]. A large proportion (86%) of the alcohol consumed is ‘homebrew’ [19] as it is more affordable in middle and low-income countries.

The prevalence of low physical activity was still low (6.2%), but higher than that of Nepal (3.4%) [20]. This could be due to a high percentage of the respondents (68.6%) residing in rural areas and 53% engaged in agriculture or other types of labor-intensive occupations. As reported elsewhere, the prevalence of low physical activity was higher in women than in men [21]. More than two-thirds of the population did not consume the recommended five or more servings of fruits and vegetables. A similar trend has been reported from Nepal (99%) and Bangladesh (91.8%) nationwide STEPS survey [20, 22]. Low consumption of fruits and vegetables could be due to seasonal availability and dietary habits. Culturally, rice is the staple food in Bhutan; 50% of the daily calorie requirement comes from rice [23].

We found nearly one-third (32.9%) of adults in Bhutan were overweight or obese. By comparison, the reported prevalence of overweight in Bangladesh, Nepal, and Thailand were 16.9%, 21%, and 28.3%, respectively [20, 22, 24]. Rapid urbanization, low nutrition literacy, sedentary lifestyle, and high carbohydrate and low protein diets are among the drivers of the global epidemic of overweight or obesity [25–27]. Similar to studies in other low-middle income countries, the prevalence of overweight or obesity increased with age, being female, engaging in certain occupations (e.g., homemakers or government employees), and residing in urban areas associated with physically inactive [20, 22, 24, 28]. Tobacco use had an inverse association with overweight or obesity. This finding was consistent with studies in Thailand and India [24, 29], which could be due to reduced food intake and the thermogenic effects of smoking [30].

In this study, we observed a positive association between fruits and vegetables intake and overweight or obesity. This could be because overweight or obese people reported consuming the recommended amount of fruits and vegetables more often. A large Australian cross-sectional study showed that women who had a higher intake of fruits and vegetables were more likely to be overweight and obese [31]. Further, a prospective cohort study from the US found that increased intake of starchy vegetables such as potatoes, peas, and corn were associated with weight gain [32]. Maize, potatoes, and rice were the staple foods in the Bhutan diet [33], and they were classed as starchy vegetables in the STEPS survey [8].

The prevalence of raised blood pressure in the adult population has increased from 26% in 2007 in Thimphu, the capital city of Bhutan [34], to 35.7%. The prevalence we reported in this study is consistent with estimates from India and Thailand [35]. Alcohol consumption is strongly associated with an increased risk of hypertension [36, 37]. Our study showed that alcohol users were 1.4 times more likely to be hypertensive than those who did not drink alcohol. Tobacco users were less likely to be hypertensive after adjusting for possible confounding factors such as alcohol use, physical activity, dietary habits, age, sex, education, occupation, marital status, and area of living. However, the association between tobacco use and hypertension was inconsistent in literature [38, 39]. Some studies failed to find a difference in the prevalence of hypertension according to smoking status [40]. Many factors contribute to the heterogeneity of the findings, including masked hypertension, time relation between tobacco consumption and measurement of blood pressure, type of tobacco use, duration, and the onset of hypertension [38, 39].

The observed prevalence of diabetes in the current study (6.4%), was similar to that of neighboring countries like India (7.3%) and Thailand (6.9%) [41, 42], higher than Nepal (3.4%) [20]. We also observed that the prevalence of diabetes increased with age, similar to overweight or obesity, one of the main risk factors for type 2 diabetes [43, 44]. Urban residents and tobacco users were also more likely to be diabetic than rural residents and non-tobacco users, respectively, consistent with findings in other studies [20, 45]. However, some common risk factors such as physically inactive, low fruits and vegetables intake, and alcohol use were not associated with diabetes. Other factors may play a role in the causes of type 2 diabetes in low-income countries [46].

### Strengths and limitations

The strengths of this study are twofold. First, we used a large nationally representative dataset collected using the WHO standardized protocol. Second, the selection bias was minimized by using the Kish method to select respondents randomly. The main limitations of the study are the cross-sectional design that does not allow to determine causality. Furthermore, the self-reported data could lead to information bias and underestimating the prevalence due to social stigma associated with tobacco use and alcohol consumption [15]. In addition, the use of a single measurement of blood pressure or glucose may be subjective to measurement error related to diagnosing hypertension or diabetes.

The findings from this nationally representative survey suggest an urgent need for the Ministry of Health, Royal Government of Bhutan to scale up the national NCDs action plan. Actions are needed to curb the NCDs epidemic, including integrating early detection and management of NCDs into primary health care, instituting NCDs risk factors surveillance into the existing Health Information Management System, and scaling up health promotion and prevention efforts to reduce the NCDs risk factors.

## Conclusion

This study provides the first comprehensive national evidence on the magnitude of modifiable risk factors, and the associations of sociodemographic and modifiable risk factors with overweight or obesity, hypertension, and diabetes. Actions are needed to scale up the implementation of the national NCDs action plan. This requires political commitment and the whole of government approach, in line with the Royal Government of Bhutan’s commitments to Gross National Happiness and Wellbeing [7]. The study also highlights that the common modifiable behavioral risk factors may not fully account for the high prevalence of overweight or obesity, hypertension and diabetes in low-middle income countries, like Bhutan. Further in-depth studies using qualitative methods are recommended to fill in the knowledge gaps.
